# Treatment of CoQ_10_ Deficient Fibroblasts with Ubiquinone, CoQ Analogs, and Vitamin C: Time- and Compound-Dependent Effects

**DOI:** 10.1371/journal.pone.0011897

**Published:** 2010-07-30

**Authors:** Luis C. López, Catarina M. Quinzii, Estela Area, Ali Naini, Shamima Rahman, Markus Schuelke, Leonardo Salviati, Salvatore DiMauro, Michio Hirano

**Affiliations:** 1 Department of Neurology, Columbia University Medical Center, New York, New York, United States of America; 2 Clinical and Molecular Genetics Unit, University College London Institute of Child Health, London, United Kingdom; 3 Department of Neuropediatrics, Charité Virchow University Hospital, Berlin, Germany; 4 Servizio di Genetica Clinica ed Epidemiologica, Department of Pediatrics, University of Padova, Padova, Italy; Hospital Vall d'Hebron, Spain

## Abstract

**Background:**

Coenzyme Q_10_ (CoQ_10_) and its analogs are used therapeutically by virtue of their functions as electron carriers, antioxidant compounds, or both. However, published studies suggest that different ubiquinone analogs may produce divergent effects on oxidative phosphorylation and oxidative stress.

**Methodology/Principal Findings:**

To test these concepts, we have evaluated the effects of CoQ_10_, coenzyme Q_2_ (CoQ_2_), idebenone, and vitamin C on bioenergetics and oxidative stress in human skin fibroblasts with primary CoQ_10_ deficiency. A final concentration of 5 µM of each compound was chosen to approximate the plasma concentration of CoQ_10_ of patients treated with oral ubiquinone. CoQ_10_ supplementation for one week but not for 24 hours doubled ATP levels and ATP/ADP ratio in CoQ_10_ deficient fibroblasts therein normalizing the bioenergetics status of the cells. Other compounds did not affect cellular bioenergetics. In *COQ2* mutant fibroblasts, increased superoxide anion production and oxidative stress-induced cell death were normalized by all supplements.

**Conclusions/Significance:**

These results indicate that: 1) pharmacokinetics of CoQ_10_ in reaching the mitochondrial respiratory chain is delayed; 2) short-tail ubiquinone analogs cannot replace CoQ_10_ in the mitochondrial respiratory chain under conditions of CoQ_10_ deficiency; and 3) oxidative stress and cell death can be counteracted by administration of lipophilic or hydrophilic antioxidants. The results of our *in vitro* experiments suggest that primary CoQ_10_ deficiencies should be treated with CoQ_10_ supplementation but not with short-tail ubiquinone analogs, such as idebenone or CoQ_2_. Complementary administration of antioxidants with high bioavailability should be considered if oxidative stress is present.

## Introduction

Coenzyme Q_10_ (CoQ_10_; ubiquinone) and its analogs have been evaluated as antioxidant agents and enhancers of mitochondrial functions in patients with mitochondrial disorders and clinical trials of neurodegenerative diseases including Parkinson disease, amyotrophic lateral sclerosis, Huntington disease, Friedreich ataxia, and Alzheimer's disease with modest or no objective benefits [1]–[6]. The use of CoQ_10_ therapy and its analogs in primary CoQ_10_ deficiency, an autosomal recessive syndrome due to defects of ubiquinone biosynthesis, could provide valuable data to evaluate the effectiveness of these compounds in restoring respiratory chain activities and preventing oxidative stress. The disorder manifests clinically with four major phenotypes: 1) an encephalomyopathy with brain involvement and recurrent myoglobinuria [7]; 2) an infantile multisystem disorder with encephalopathy usually associated with nephropathy and variable involvement of other organs [8], [9]; 3) ataxic syndrome with cerebellar atrophy [10], [11]; and 4) an isolated myopathy [12], [13].

**Figure 1:**
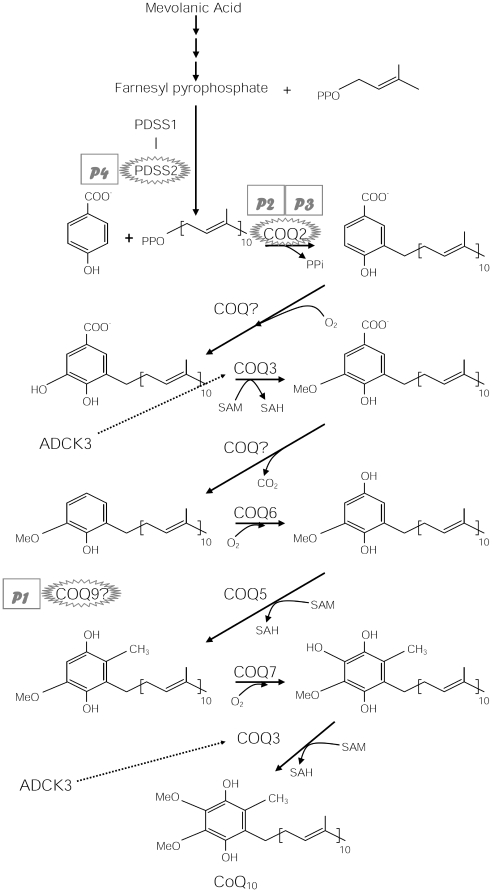
CoQ_10_ biosynthesis pathway. CoQ_10_ is composed of a benzoquinone and a decaprenyl side chain. PDSS2 is the second subunit of decaprenyl diphosphate synthase, a heterotretameric enzyme that catalyzes the formation of the decaprenyl side chain. COQ2 or *para*-hydroxybenzoate (PHB)-polyprenyl transferase catalyzes the condensation reaction of PHB and decaprenyl diphosphate. The function of COQ9 is still unknown. The mutant fibroblasts used in this study harbor mutations in COQ9, p.244R>X (P1) [18], COQ2, p.197R>H, p.228N>S (P2) [14] and p.297Y>C (P3) [17], and PDSS2, p.322Q>X, p.382S>L (P4) [20].

Molecular defects in genes encoding CoQ_10_ biosynthetic proteins have been reported in 18 patients. Four patients improved with CoQ_10_ supplementation [9], [14]–[17], five died before or during the treatment, and 9 had no definite response [14], [15], [18]–[22]; it is therefore difficult to reach definitive conclusions about the effectiveness of CoQ_10_ supplementation in primary CoQ_10_ deficiencies. To better understand the pathogenesis of CoQ_10_ deficiency, we have characterized the bioenergetics and oxidative stress in *PDSS2* and *COQ2* mutant fibroblasts, and have demonstrated that severe CoQ_10_ deficiency caused marked defects of ATP synthesis without oxidative stress whereas milder CoQ_10_ deficiency produced reactive oxygen species (ROS) and oxidation of proteins and lipids [23]. Here, we evaluate the *in vitro* effects of CoQ_10_ supplementation on the bioenergetics and oxidative stress status of CoQ_10_ deficient fibroblasts with mutations in *PDSS2*, *COQ2*, and *COQ9* (Fig. 1). In addition, because CoQ_10_ analogs and vitamin C are being used in clinical trials based on their antioxidant properties, we concurrently evaluated the effect of CoQ_2_, idebenone, and vitamin C.

## Results

### Cellular CoQ_10_ levels after treatment with compounds for 24 hours

**Figure 2:**
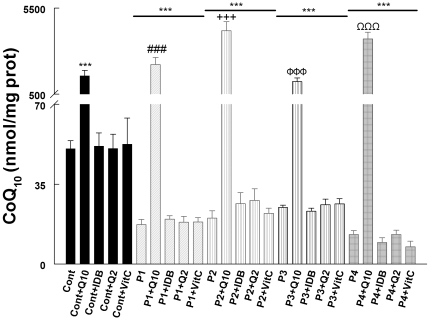
Cellular CoQ_10_ levels after 24 hours of treatment. Cultured skin fibroblasts from controls and CoQ_10_ deficiency patients were treated with 5 µM CoQ_10_, idebenone, CoQ_2_ or vitamin C. After 24 hours, fibroblasts were collected and cellular CoQ_10_ were determined by EQ-HPLC. Results are expressed as a mean ± SD of three experiments. P1 = *COQ9* mutant; P2 =  *COQ2* mutant; P3 =  *COQ2* mutant; P4 = *PDSS2* mutant. ***P<0.001 compared with untreated control; ###*P*<0.001 compared with untreated P1; +++*P*<0.001 compared with untreated P2; φφφ *P*<0.001 compared with untreated P3; ΩΩΩ *P*<0.001 compared with untreated P4.

Fibroblasts from the four patients with different molecular defects in the CoQ_10_ biosynthetic pathway used in this study showed significantly decreased levels of CoQ_10_ relative to controls (*P*<0.001) (Fig. 2). When control and patients' cells were treated for 24 h with 5 µM of CoQ_10_, cellular levels of ubiquinone increased significantly in all cells (*P*<0.001), resulting in values 20–85-fold higher than in control cells (Fig. 2). When the cells were treated with idebenone, CoQ_2_, or vitamin C, cellular levels of CoQ_10_ remained unchanged (Fig. 2).

### Bioenergetic status: 24 hours versus 1 week of treatment

**Figure 3:**
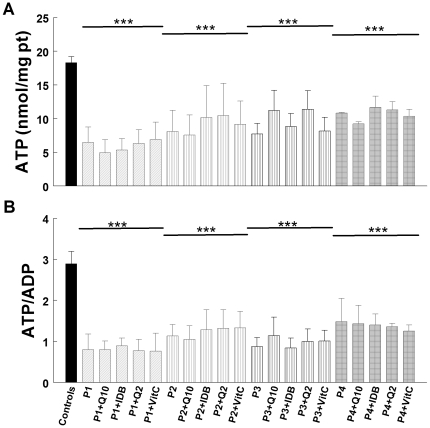
Bioenergetic status of cells after 24 hours of treatment. Cultured skin fibroblasts from controls and CoQ_10_ deficient patients were treated with 5 µM CoQ_10_, idebenone, CoQ_2_, or vitamin C. After 24 h, fibroblasts were collected and cellular ATP and ADP were determined by UV-HPLC. Data are represented as nmol of ATP/mg prot (**A**) and ATP/ADP ratio (**B**). Results are expressed as mean ± SD of three experiments. P1 = *COQ9* mutant; P2 =  *COQ2* mutant; P3 =  *COQ2* mutant; P4 = *PDSS2* mutant. ***P<0.001 compared with untreated control.

**Figure 4:**
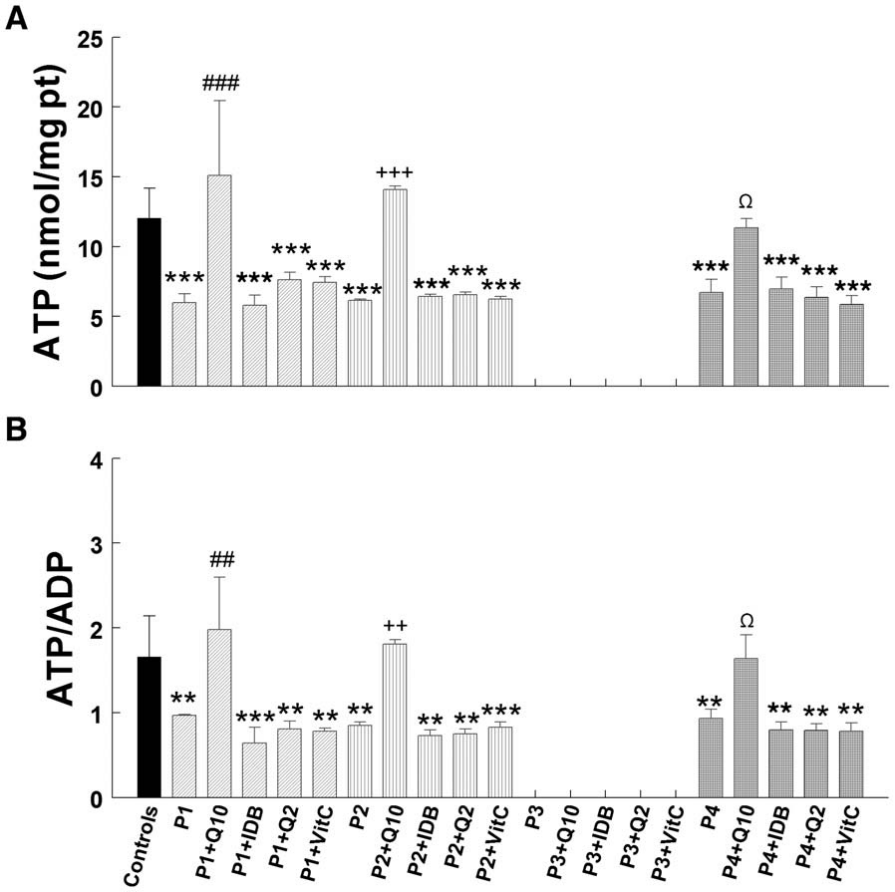
Bioenergetic status of cells after 1 week of treatment. Cultured skin fibroblasts from controls and CoQ_10_ deficient patients were treated with 5 µM CoQ_10_, idebenone, CoQ_2_, or vitamin C. After 1 week, fibroblasts were collected and cellular ATP and ADP were determined by UV-HPLC. Data are represented as nmol of ATP/mg prot (**A**) and ATP/ADP ratio (**B**). Results are expressed as mean ± SD of three experiments. P1 = *COQ9* mutant; P2 =  *COQ2* mutant; P3 =  *COQ2* mutant; P4 = *PDSS2* mutant. ***P<0.001 and **P<0.01 compared with untreated control; ##*P*<0.01 and ###*P*<0.001 compared with untreated P1; ++*P*<0.01 and +++*P*<0.001 compared with untreated P2; Ω *P*<0.05 and ΩΩ *P*<0.01 compared with untreated P4.

The four CoQ_10_ deficient fibroblasts showed significant decreases in cellular ATP levels (*P*<0.001) (Fig. 3A) and ATP/ADP ratios (*P*<0.001) (Fig. 3B). Treatment with 5 µM CoQ_10_, idebenone, CoQ_2_, or vitamin C for 24 h did not increase ATP levels or ATP/ADP ratios. However, treatment with 5 µM CoQ_10_ for 1 week increased significantly levels of cellular ATP in P1 (*P*<0.001), P2 (*P*<0.001), and P4 (*P*<0.05) to normal levels (Fig. 4A). At the same time, ATP/ADP ratios were significantly increased in P1 (*P*<0.01), P2 (P<0.01), and P4 (*P*<0.05) (Fig. 4B) thus bringing to normal the bioenergetics status of the cells. In contrast, idebenone, CoQ_2_, and vitamin C treatment failed to alter ATP levels or ATP/ADP ratios (Fig. 4).

### The effect of compound administration on superoxide anion levels: 24 hours versus 1 week of treatment

**Figure 5:**
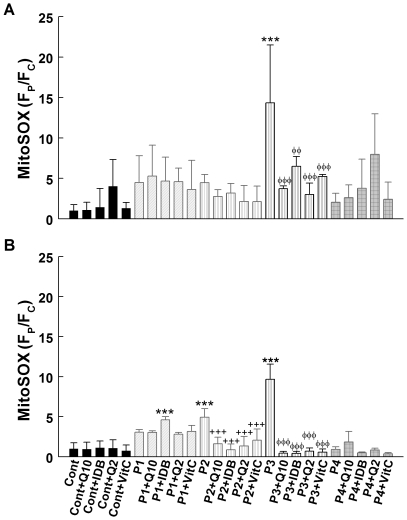
Superoxide anion production in cells treated with antioxidant compounds for 24 hours (A) or 1 week (B). Cultured skin fibroblasts from controls and CoQ_10_ deficient patients were treated with 5 µM CoQ_10_, idebenone, CoQ_2_, or vitamin C. After 24 h (**A**) or 1 week (**B**), fibroblasts were collected and superoxide anion generation was assessed by MitoSOX staining and quantified by flow cytometry The Y axis represents the fluorescence intensity of the sample relative to the fluorescence intensity of the control sample (F_P_/F_C_), after subtracting the background intensity; the X axis represent the sample. 20,000 cells were analyzed in every experiment. Results are expressed as mean ± SD of three experiments. P1 = *COQ9* mutant; P2 =  *COQ2* mutant; P3 =  *COQ2* mutant; P4 = *PDSS2* mutant. ***P<0.001 compared with untreated control; ##*P*<0.01 and ###*P*<0.001 compared with untreated P1; +++*P*<0.001 compared with untreated P2; φφ *P*<0.01 and φφφ *P*<0.001 compared with untreated P3.

After incubation for 24 h in galactose medium supplemented with *dialyzed* FBS, P3 cells showed increased MitoSOX Red stain indicating elevated levels of superoxide anions (*P*<0.001) (Fig. 5A). Under the same culture conditions, the other three CoQ_10_ deficient fibroblasts did not show any significant changes in MitoSOX stain. After 24 h of treatment with CoQ_10_, idebenone, CoQ_2_, or vitamin C, superoxide anion levels decreased significantly in P3 cells (*P*<0.01) (Fig. 5A). Cells from controls and from the three other patients did not show significant changes in MitoSOX stain after 24 hours of treatment with any of the four compounds; however, there was a trend towards increased superoxide anions in P4 cells treated with CoQ_2_ for 24 h (Fig. 5A).

After one week of incubation in galactose medium plus *dialyzed* FBS, P2 and P3 cells showed increased MitoSOX Red staining (Fig. 5B). After 1 week of treatment with CoQ_10_, idebenone, CoQ_2_, or vitamin C, superoxide anion levels were decreased significantly in both P2 and P3 cells (*P*<0.001) (Fig. 5B).

### The effect of compound administration on cell death: 24 hours versus 1 week treatment

**Figure 6:**
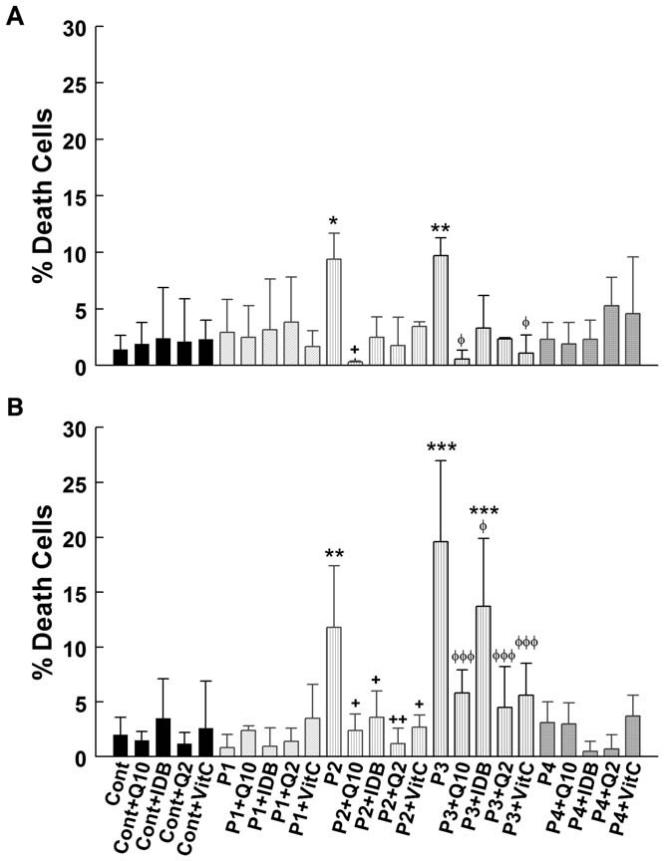
Dead cells after 24 hours (A) or 1 week of treatment (B). Cultured skin fibroblasts from controls and CoQ_10_ deficiency patients were treated with 5 µM CoQ_10_, idebenone, CoQ_2_, or vitamin C. After 24 h (**A**) or 1 week (**B**), fibroblasts were collected and Trypan blue assays were performed. Results are expressed as mean ± SD of three experiments. P1 = *COQ9* mutant; P2 =  *COQ2* mutant; P3 =  *COQ2* mutant; P4 = *PDSS2* mutant. *P<0.05, **P<0.01 and ***P<0.001 compared with untreated control; +*P*<0.05 and ++*P*<0.01 compared with untreated P2; φ*P*<0.05 and φφφ *P*<0.001 compared with untreated P3.

In adherent cells, cell death was significantly higher in untreated P2 and P3 cells than in control cells (*P*<0.05 and *P*<0.01, respectively) (Fig. 6A). Cell death was significantly reduced in both P2 and P3 treated for 24 h with CoQ_10_ (*P*<0.01) or idebenone (*P*<0.05). Vitamin C also reduced cell death in P3 cells (*P*<0.05) (Fig. 6A). Control, P1, and P4 cells showed similar proportions of dead cells with and without treatment (Fig. 6A).

Untreated P2 and P3 cells also showed significantly higher cell death than controls when incubated for 1 week in galactose medium supplemented with *dialyzed* FBS (P<0.01 and P<0.001, respectively) (Fig. 6B). Cell death was reduced in P2 cells by 24 h treatment with CoQ_10_ (*P*<0.05), idebenone (*P*<0.05), CoQ_2_ (*P*<0.01), or vitamin C (*P*<0.05); and in P2 cells by one week treatment with CoQ_10_ (*P*<0.001), idebenone (*P*<0.05), CoQ_2_ (*P*<0.001), or vitamin C (*P*<0.001) (Fig. 6B). While the P3 cell death levels were significantly reduced by treatment with idebenone for 1 week (*P*<0.05), they were still significantly higher than those of control cells (*P*<0.001) (Fig. 6B).

Because we observed abundant floating *COQ2* mutant cells cultured in galactose medium with *dialyzed* FBS, we performed Trypan blue staining of P2 cells and found that 15% of the cells were dead after 24 h and 63% were dead after 1 week. Percentages of floating dead cells were significantly decreased after one week of treatment with CoQ_10_ (36% dead cells), CoQ_2_ (45% dead cells), or idebenone (43% dead cells).

## Discussion

Over the last 4 years, studies of CoQ_10_ deficient patients have focused on two major issues: identifying the molecular genetic basis of ubiquinone deficiencies and characterizing pathogenic mechanisms [24]. We identified the two first mutations in genes encoding proteins required for CoQ_10_ biosynthesis, *COQ2* [17] and *PDSS2* [20], in patients with primary CoQ_10_ deficiency. Our studies of cultured fibroblasts from these patients revealed that both CoQ_10_ biosynthetic disorders caused bioenergetic defects, but only *COQ2* mutant fibroblasts showed increased ROS production and signs of oxidative stress [23]. In the present study, we have evaluated the *in vitro* efficacy of CoQ_10_ supplementation in normalizing the bioenergetic status and the oxidative balance in fibroblasts of CoQ_10_ deficient patients harboring mutations in *PDSS2*, *COQ2*, and *COQ9* [14], [17], [18], [20], [23]. We chose a final concentration of 5 µM CoQ_10_ because this is the approximate concentration reached in the plasma of patients treated with oral supplementation of CoQ_10_ [3], [6], [25]–[27]. We have also evaluated the efficacy of short-tail ubiquinone analogs (idebenone and CoQ_2_), which are less lipophilic, and vitamin C, a well-known hydrophilic antioxidant. It is noteworthy that 5 µM concentrations of ubiquinone analogs exceed the EC_50_ of idebenone (∼0.4–0.5 µM) and CoQ_2_ (∼0.03 µM) in cultured fibroblasts under oxidative stress [28], [29].

After 24 h of 5 µM CoQ_10_ supplementation, cellular CoQ_10_ levels increased dramatically. The magnitudes of the increases were independent of the molecular defects of the cells since both control and patients' fibroblasts showed similar degrees of CoQ_10_ uptake. However, after 24 h of CoQ_10_ treatment, none of the cell lines showed significant improvement in ATP levels or in ATP/ADP ratios, which are markers of respiratory chain function. In contrast to our results, López-Martín and colleagues [30] noted normalization of mitochondrial complexes I+III and II+III activities in *COQ2* mutant fibroblasts after 24 h of 10 µM CoQ_10_ supplementation. Paradoxically, however, the same authors using the same *COQ2* mutant cells and other genetically undefined CoQ_10_ deficient fibroblasts found that the activities of complex II+III activities increased only slightly and remained below control values after 72 hours of 100 µM CoQ_10_ supplementation [31]. This discrepancy may be due to the fact that in the second study, but not in the first, respiratory chain enzyme activities were normalized to the activity of citrate synthase [31], a marker of mitochondrial mass [32]. In another study, embryonic cells from *COQ7* knockout mice treated with 25 µM of water-soluble CoQ_10_ for 5 h only showed slight increase of ATP [33]. The negligible effects of CoQ_10_ supplementation on the bioenergetic status of CoQ_10_ deficient cells can be explained by the strong lipophilic nature of CoQ_10_, which accumulates in membranes reaching saturated concentrations [34], [35]. Thus, exogenous CoQ_10_ is mainly distributed in lysosomes, endoplasmic reticulum, and plasma membrane and only a small proportion (∼11%) reaches the mitochondria [36], [37]. In addition, much of the CoQ_10_ in mitochondria is likely to be trapped in the outer membrane, not available to the respiratory chain, which is located in the mitochondrial inner membrane [38].

Because 5 µM CoQ_10_ did not increase the ATP levels after 24 h of supplementation, we tried two other strategies: 1) increasing the duration of CoQ_10_ supplementation to 1 week; and 2) using less-lipophilic ubiquinone analogs. In marked contrast to treatment for 24 h of CoQ_10_, incubation of ubiquinone-deficient fibroblasts for 1 week with 5 µM CoQ_10_ increased ATP levels and ATP/ADP ratios significantly, indicating normalization of the bioenergetic status. Similar to these results, yeast coq mutants show inefficient uptake of exogenous CoQ_6_ to the mitochondrial inner membrane, which is reflected in a low succinate cytochrome *c* reductase activity after 2–15 µM CoQ_6_ supplementation for 48 h [37], [39]. The complete rescues of growth of the yeast coq mutants' supplemented with 15 µM CoQ_6_ were only possible after 6–8 days [37], [39], [40]. These results suggest that the pharmacokinetic constraints of CoQ_10_ in reaching the mitochondrial respiratory chain are key limiting factors in determining its efficacy in CoQ_10_ deficient patients. Furthermore, an *in vivo* study of *PDSS2* mutant mice treated for 4 months with 100 mg/kg body weight (b.w.)/day or 200 mg/kg b.w./day of water-soluble CoQ_10_ showed that only the highest dose of CoQ_10_ improved the renal function in these CoQ deficient animals [41]. Thus, the dose of CoQ_10_ is another important factor influencing the effectiveness of CoQ_10_ supplementation in ubiquinone deficient patients. Nevertheless, the gold standard to evaluate the effectiveness of CoQ_10_ treatment in ubiquinone deficiency is *in vivo* measurements of mitochondrial function, bioenergetics, and oxidative stress.

In contrast to CoQ_10_, short-tail ubiquinone analogs and vitamin C failed to increase ATP levels and ATP/ADP ratio in patients' cells after both 24 h and 1 week treatment. These results are in agreement with previous studies showing that hydrophilic ubiquinone analogs (CoQ_2_ and idebenone) or mitochondrial-targeted ubiquinone (MitoQ) are less efficient than hydrophobic ubiquinone analogs in enhancing energy production by the mitochondrial respiratory chain [42]–[44], since the specific effects of ubiquinones depend on their interaction with hydrophilic and hydrophobic (or physiological) binding sites [42], [43]. Accordingly, hydrophobic and hydrophilic ubiquinone analogs are not interchangeable. Experience with two patients with CoQ_10_ deficiency and cerebellar ataxia due to *ADCK3*/*CABC1* mutations is relevant. Both patients were treated with CoQ_10_ (5–10 mg/kg/b.w.) [21], [45]. One patient, who also had exercise intolerance and hyperlactatemia, improved after 3 months of treatment whereas the other, who had only ataxia, did not benefit from 3 years of therapy. The ataxia did not improve in either patient and for this reason CoQ_10_ was replaced with low-dose idebenone in both patients (5–10 mg/kg/b.w.). However, their symptoms worsened, prompting reinstitution of CoQ_10_ therapy [21], [45]. Based on our *in vitro* results showing improvements of bioenergetics by ubiquinone in CoQ_10_ deficient cells, we postulate that CoQ_10_ therapy improved the first patient's exercise intolerance and may have stabilized symptoms in the second patient because of enhanced ATP production. The lack of improvement of cerebellar symptoms may be due to poor transfer of CoQ_10_ across the blood brain barrier, irreversible structural alterations in the cerebellum, or both factors [36], especially considering the low dose used (5–10 mg/kg/b.w.). Our finding that high doses of idebenone *in vitro* failed to increase ATP levels in CoQ_10_ deficient fibroblasts or the low-dose used for therapy may explain ineffectiveness of idebenone therapy compared to CoQ_10_ treatment in both patients.

Because oxidative stress is another important pathogenic factor in CoQ_10_ deficiencies, we evaluated also the effect of CoQ_10_ and three other antioxidants on superoxide anion production. After both 24 h and after 1 week of treatment, we noted that all four compounds significantly reduced MitoSox staining in P3 cells (the cell line with the most prominent MitoSox fluorescence). Reduction of superoxide anion levels correlated with decreased cell death, suggesting that ROS generation and oxidative damage are the main causes of death in *COQ2* mutant fibroblasts. This notion is supported by our observation that P3 *COQ2* fibroblast line had the highest levels of superoxide anion production and protein oxidation and showed the most robust features of apoptosis and the highest proportion of cell death [46]. In contrast to *COQ2* mutant fibroblasts, *COQ9* and *PDSS2* mutant fibroblast do not have increased ROS, a difference that may be related to the disparate CoQ-dependent flow of electrons in the mitochondrial respiratory chain in the different mutant cell lines. As we recently reported, 10–15% residual CoQ_10_ are not associated with significant ROS production, whereas 30–50% residual CoQ_10_ is accompanied by increased ROS production and cell death [23], [46]. Interestingly, there was a trend towards increased ROS production in P4 cells supplemented with CoQ_2_ for 24 h and in P1 cells supplemented with idebenone for 1 week. In contrast, idebenone decreased ROS levels in the other 3 cell lines after one week of treatment. Our data suggest that these compounds may have pro-oxidant and anti-oxidant properties under certain *in vitro* conditions [47] and confirm in cultured fibroblasts observations previously reported in isolated mitochondria [48].

In summary, our *in vitro* study tackles important issues regarding treatment of CoQ_10_ deficiency and, more in general, of mitochondrial diseases. First, the prolonged pharmacokinetics of CoQ_10_ to reach the mitochondrial respiratory chain is critical to the restoration of energy status of human CoQ_10_ deficient cells. This may explain the delayed clinical response of CoQ_10_ deficiency patients to oral supplementation with CoQ_10_ [16] and suggest that high doses of CoQ_10_ are needed. Second, short tail ubiquinone analogs cannot substitute for CoQ_10_ in the mitochondrial respiratory chain of human CoQ_10_ deficient cells revealing the importance of the decaprenyl tail. Third, oxidative stress and cell death can be attenuated by the administration of lipophilic and hydrophilic antioxidants.

Thus, our results confirm that in CoQ_10_ deficient patients: (i) early treatment based on early diagnosis is critical to maximize the efficacy of ubiquinone supplementation; (ii) short-tail ubiquinone analogs (i.e. idebenone or CoQ_2_) are not suitable substitutes for CoQ_10_; and (III) complementary administration of antioxidants with high bioavailability may be helpful in CoQ_10_ deficiency patients. Further studies on the pathogenesis of CoQ_10_ deficiency in patients with different molecular defects and in animal models will lead to more rational and improved therapies.

## Materials and Methods

### Ethics Statement

Skin biopsies, to generate cultured fibroblasts, were performed after obtaining written informed parental consents under study protocols approved by the Institutional Review Boards of the Columbia University Medical Center, University of Padova, and Charité University Hospital as well as Local Research Ethics committees of Great Ormond Street Hospital for Children NHS Trust and Institute of Child Health. The informed consents and clinical studies were in compliance with the principles expressed in the Declaration of Helsinki.

### Cells culture and treatment

All experiments were performed in human skin fibroblasts from 5 controls and 4 CoQ_10_ deficient patients (Fig. 1): P1 with a homozygous mutation in *COQ9* (p.R244X) [18]; P2 with compound heterozygous mutations in *COQ2* (p.R197H and p.N228S) [14]; P3 with a homozygous mutation in *COQ2* (p.Y297C) [17]; and P4 with compound heterozygous mutations in *PDSS2* (p.Q322X and p.S382L) [20]. Cells were grown in RPMI high-glucose medium supplemented with 10% fetal bovine serum, 5 ml MEM non-essential amino acids, 1 ml fungizone, and 5 ml penicillin-streptomycin until 80% confluent.

For the 24 h treatment experiments, 75% confluent cells were grown in RPMI-1640 glucose-free medium with 10% *dialyzed* fetal bovine serum, 25 mM HEPES, 1.5 mM Glutamax, 25 mM galactose, 1 ml fungizone, and 5 ml penicillin-streptomycin for 2 days. Twenty-four hours before the experiments, cells were incubated in fresh medium supplemented with idebenone (Santhera Pharmaceuticals, Liestal, Switzerland), CoQ_2_ (Sigma–Aldrich Corp., St. Louis, MO, USA), CoQ_10_ or vitamin C (Sigma-Aldrich Corp., St. Louis, MO, USA) (5 µM final concentration).

For the 1 week treatment experiments, 60% confluent cells were incubated for 7 days in RPMI-1640 glucose-free medium with 10% *dialyzed* fetal bovine serum, 25 mM HEPES, 1.5 mM Glutamax, 25 mM galactose, 1 ml fungizone, 5 ml penicillin-streptomycin and one of the following: idebenone, CoQ_2_, CoQ_10_, or vitamin C (5 µM final concentration).

Regular FBS contains 85–245 µg glucose/ml and *dialyzed* FBS less than 5 µg glucose/ml. Because our experiments required glucose-free media supplemented with galactose to force energy production through oxidative phosphorylation rather than glycolysis [23], [49], [50], we used 10% *dialyzed* FBS. This was particularly important for the 24 h treatment experiments since cells can use the glucose contained in 10% regular FBS during the first 24–48 h.

Idebenone, CoQ_2_, CoQ_10_, and vitamin C were dissolved in *dialyzed* fetal bovine serum prior to addition to culture medium.

All cells culture reagents were obtained from Invitrogen Corp. (Carlsbad, CA, USA). Patients and control cell lines at passage 7–10 were grown to replicate every experiment at least 3 times.

### Cellular CoQ_10_ levels

One week after treatment, fibroblast pellets were collected and washed 4 times with phosphate buffered saline (PBS). After the fourth wash, no CoQ_10_ was detected in the PBS supernatant. CoQ_10_ from fibroblasts was extracted in a hexane:ethanol mixture [20]. The lipid component of the extract was separated by HPLC on a reverse phase Symmetry® C18 3.5 µm, 4.6×150 mm column (Waters), using a mobile phase consisting of methanol, ethanol, 2-propanol, acetic acid (500∶500∶15∶15) and 50 mM sodium acetate at a flow rate of 0.9 ml/min. The electrochemical detector consisted of an ESA Coulochem II with the following setting: guard cell (upstream of the injector) at +900 mV, conditioning cell at –600 mV (downstream of the column), followed by the analytical cell at +500 mV. CoQ_10_ concentration was estimated by comparison of the peak area with those of standard solutions of known concentration [20].

### Mitochondrial bioenergetics assessment

Fibroblasts cultured under glucose-free media supplemented with galactose possess ATP and ADP pools that are predominantly dependent on the oxidative phosphorylation [23], [49]; therefore, using these culture conditions, ATP levels and ATP/ADP ratio are reliable markers of oxidative phosphorylation and correlate with the rates of ATP synthesis in CoQ_10_ deficient cells, as reported [23]. To determine the adenine nucleotides levels, cells were grown in 15-cm-diameter plates to 90% confluency and the adherent cells were collected in ice-cold PBS using a scraper. After centrifugation at 3,000 *g* for 3 min at 4°C, pellets were suspended in 200 µl of ice-cold 0.5 M perchloric acid, vortexed for 30 s, and centrifuged at 11,000 *g* for 10 min at 4°C. Pellets were stored at −80°C for protein measurement. Adenine nucleotides were measured in the supernatants injected into an Alliance HPLC (Waters Corporation, Milford, MA, USA) with an Alltima C18 NUC reverse-phase column (Alltech Associates, Deerfield, IL, USA) [23]. Adenine nucleotide levels were expressed in nmol/mg protein. Because of poor growth of P3 cells, the 1 week experiments were performed only in P1, P2 and P4 fibroblasts.

### Oxidative stress analyses

Previously, we demonstrated that oxidative stress can be detected by MitoSOX Red assay, lipid peroxidation assay, and carbonyl groups detection in CoQ_10_ deficient cells due to *COQ2* mutation [23]; and results of the three assays were highly concordant [23]. Here, to evaluate the effect of the proposed treatments in those and others CoQ_10_ deficient cells, we estimated mitochondrial matrix oxidant levels in control and mutant fibroblasts using MitoSOX Red, a fluorochrome specific for mitochondrial matrix reactive oxygen species (ROS) burden (Molecular Probes, Invitrogen Corp., Carlsbad, CA, USA) as described [23], [51]. Approximately 1×10^6^ adherent cells (90% confluence) were trypsinized, incubated with 5 µM MitoSox for 20 min at 37°C in the dark, washed twice with PBS and re-suspended in 500 µl of PBS. Cytofluorometric analysis was performed using a FACSCalibur cell analyzer equipped with a 488 nm At-kt laser and fluorescence was measured using the FL2 channel. Fluorescence was background subtracted and normalized to the signal of control values (F_P_/F_C_). Data were acquired using Cell Pro Quest and analyzed using Flowjo software (Becton Dickinson, NJ, USA). Before to start with the set of experiments for the cytofluorometric analysis, the MitoSOX staining was evaluated and monitored by fluorescence microscopy (IX70 inverted system microscope, Olympus, Tokyo, Japan). Additionally, a control cell line treated with 0.5 µM antimycin A for 15 h before the MitoSOX staining was used as a positive control for increased ROS production [23].

### Oxidative stress-induced death cells studies

Measurement of cell viability was performed with the Trypan blue exclusion method. Numbers of living and dead adherent cells were determined by light microscopy using a hemocytometer. We also determined the proportion dead non-adherent P2 cells. Healthy nuclei from viable cells appeared round and phase bright, whereas nuclei from dead or dying cells appeared blue and irregularly shaped. All cells were counted and results were expressed as percentage of dead cells relative to total cells [52].

### Statistical analysis

Control data are expressed as mean ± SD of 5 different samples in triplicate experiments. Patients' data are expressed as mean ± SD of triplicate experiments. One-way analysis of variance (ANOVA) followed by Student-Newman-Keuls multiple comparisons test was used. A P value of less than 0.05 was considered statistically significant.
